# Sociodemographic Correlates of Integrated Maternal, Newborn, and Child Health Services Utilization Among Childbearing Mothers in Nigeria

**DOI:** 10.7759/cureus.57806

**Published:** 2024-04-08

**Authors:** Chima C Igbokwe, Cylia Iweama, Aminu Igwe, Lawreta I Abugu, James T Ihongo, Jacinta E Ugbelu, Ugbedeojo Adejo Sule

**Affiliations:** 1 Department of Human Kinetics and Health Education, University of Nigeria, Enugu, NGA; 2 General Practice, Hull University Teaching Hospitals NHS Trust, Hull, GBR; 3 Department of Community Health, NKST College of Health Technology, Nkar, NGA; 4 Global Food, Nutrition and Health, University of Bayreuth, Bayreuth, DEU

**Keywords:** integrated maternal newborn and child health, place of residence, healthcare correlates, sociodemographic factors, health service utilization

## Abstract

Background

Some social and demographic factors might limit the ability of childbearing mothers (CBMs) to use healthcare services for themselves and their children, thereby exposing them to maternal, infant, and child morbidity and mortality. This study aimed to investigate the sociodemographic correlates of the utilization of integrated maternal, newborn, and child health (IMNCH) services by CBMs in Benue State, Nigeria.

Methodology

A community-based, correlational survey study was conducted among a random sample of 1,200 CBMs. Face-to-face interviews were conducted using a pretested, structured questionnaire, the Integrated Maternal, Newborn, and Child Health Services Utilization Questionnaire (IMNCHSUQ), and study participants were chosen using a multistage sampling procedure. Of the IMNCHSUQ issued, only 896 copies had complete data, which were used for the analysis. The data collected were analyzed using SPSS version 25.0 (IBM Corp., Armonk, NY, USA). The data were evaluated using the mean, standard deviation, and point-biserial correlation, and the null hypotheses were tested using linear regression at the 0.05 level of significance.

Results

The majority of the CBMs were married (79.7%) and unemployed (66.0%) while the predominant age group was between 15 and 24 years (42.7%). CBMs had a high level of utilization of IMNCH services (x= 3.30, SD = 0.94). The sociodemographic factors studied had little or no influence on the utilization of IMNCH services among CBMs. The most significant demographic predictor was the place of residence. Marital status, educational level, parity, and age were also found to be significant predictors in a few services.

Conclusions

Uneven distribution of IMNCH centers may be a major cause of the failure of CBM’s lack of utilization of IMNCH services, especially in rural settlements with sparse distribution of facilities.

## Introduction

Maternal and child health services have become one of the most significant public health initiatives that impact both national and international well-being. This is because pregnancy, successful delivery, and the subsequent postpartum phase impact every individual, family, and community at some point [1]. Maternal and child mortality is declining globally, according to current estimates, but regional data reveal that it is still quite high in low- and middle-income countries, where it accounts for 86% of all recorded maternal fatalities worldwide [1,2]. The lifetime chance of dying during pregnancy, labor, or the postpartum period is 1 in 22, with Nigeria having the highest estimated maternal fatalities worldwide and the worst newborn mortality deaths in Africa [3]. The use of health services by women and the sufficiency of high-quality supplies of maternity and pediatric healthcare at various levels of care have been linked to several issues [4]. Therefore integrated maternal, newborn, and child health (IMNCH) services mainly aim to better equip expectant women to have successful pregnancies, deliveries, and childcare experiences [5,6].

The goal of the IMNCH services is to lower rates of maternal, newborn, and child morbidity and death in the community, particularly in rural areas, by improving access to and usage of maternal and child health services. By providing health education and raising knowledge about maternal health, the IMNCH program aids in the early identification of mothers at high risk of morbidity and death during the prenatal and postnatal periods [5,6]. Although Nigeria has made substantial progress in personnel and infrastructure development, the country’s average maternal death rate still ranks among the highest worldwide, with considerable state-to-state differences. However, recent data has demonstrated that, due to prevailing sociodemographic constraints, these advancements in infrastructure and staff are still insufficient for supply and remain underutilized [4,7,8].

The utilization of health services can be improved to reduce maternal mortality in Nigeria by raising awareness regarding the utilization of maternal health services, bringing it closer to the mothers, making it more affordable, and improving its quality [5,6]. Utilization of IMNCH services has been found to be highly impacted by childbearing mothers’ (CBMs) age, place of residence, parity, marital status, and educational level [7,8]. Early childbearing has been linked to a higher risk of morbidity, including ectopic pregnancy, preterm labor, premature rupture of the membranes, vesicovaginal fistulae, and even maternal death [9]. Low-income communities and lack of education constitute factors responsible for restricted access to information about antenatal care services (ANCs) and may lead to maternal morbidity and mortality [7,8]. The adoption of folkloric or traditional techniques, which are inefficient and expose women to unexpected pregnancies and unsafe abortions, results from inadequate education and perceived fear of the negative effects of contraceptives, causing reductions in the utilization of family planning services [8,10].

State-to-state differences exist in the use of IMNCH services, and these factors all contribute to the average national maternal death rate in Nigeria [4]. Recent studies have shown a high utilization of IMNCH services among CBMs in Benue state [10,11]. Despite this, the maternal mortality ratio (MMR) in Benue state remains as high as 1,189 per 100,000 population, making the state one of the highest contributors to the national average of 576 deaths per 100,000 [10]. Increased awareness drives, infrastructural development, and cash rewards to CBMs comprise some of the government’s responses to the burden of MMR in Benue state [12,13].

This study was conducted owing to the lack of literature on the sociodemographic factors affecting the utilization of IMNCH services in Benue State. Therefore, this study aimed to investigate the sociodemographic correlates of the utilization of IMNCH services by CBMs in Benue State, Nigeria.

## Materials and methods

Study setting

The area of the study is Benue State, Nigeria. It is one of the 36 states of Nigeria and is located in the north-central area. The 2016 prediction puts its population at 5,741,800 [14]. There is at least one operational healthcare facility in each of the Local Government Areas (LGAs), rural and urban. The healthcare centers where CBMs receive care are either government-run or privately owned.

Study design

A retrospective, correlational, survey research design was used in this study.

Sample size determination and sampling procedure

Using a percentage method consistent with the literature, the study’s sample size of 1,200 CBMs was established [15]. Subsequently, 10% of the required sample size was added to allow for possible non-response or other sources of data loss, in addition to the 5% significance level and error margin.

The sample selection was done using the multistage sampling process. Using a straightforward random selection method of voting without replacement, two senatorial districts were chosen in the first stage from among the three senatorial districts in Benue State. Then, from the two senatorial districts that were chosen, three LGAs each were chosen using a straightforward random sample method of balloting without replacement. As a result, six LGAs were included in the research. Using purposive sampling, 48 primary health centers (eight from each LGA) were chosen. Additionally, due to the large number of IMNCH services they offer, the Federal Medical Centre and Benue State University Teaching Hospital in Makurdi were specifically chosen for the study. Convenience sampling was utilized in the fourth stage to choose 1,200 CBMs (24 from each institution) who agreed to participate in the research. The women were chosen from the health facilities on ANC visits and vaccination days.

Data collection tool and analysis

For gathering data, the Integrated Maternal, Newborn, and Child Health Services Utilization Questionnaire (IMNCHSUQ) was employed. The IMNCHSUQ comprises three sections, i.e., A, B, and C, and contains 18 items. Seven questions in Section A asked about the demographic characteristics of the CBMs. Six questions in Section B were designed to gather data on the extent to which CBMs utilized IMNCH services. Five items in Section C addressed cultural variables influencing women’s use of IMNCH services. A four-point Likert-type response option was used for Section B of the questionnaire: always (4 points), occasionally (3 points), seldom (2 points), and never (1 point). The questionnaire items that most accurately represented the respondents’ level of use during the previous 12 months had to have a tick (√) placed next to them. Five elements in Section C addressed cultural aspects that affect CBMs’ use of IMNCH services. The following are some of the questions: Is it bad to have a cesarean section? Is it bad to visit health facilities during ANC, birth, or in the event of a complication? What is the optimal location for delivery? Are puerperal fever and psychosis following delivery brought on by evil spirits? The questions were created with cultural norms, values, taboos, and beliefs in mind. The dichotomous response options for this section were “Yes” or “No.” The participants needed to mark the items (√) that they believed affected their utilization of IMNCH services.

Five experts from the researchers’ institution were given a draft copy of the instrument, together with the study’s goal, research questions, and hypotheses, to establish the instrument’s face validity. For Sections B and C of the IMNCHSUQ, the split-half method and Cronbach’s alpha were used to calculate the reliability index, respectively. For the full scale, reliability indices of 0.97 and 0.84, respectively, were attained. As a result, the device was considered trustworthy [15]. The researcher, along with five research assistants, distributed 1,200 copies of the questionnaire to each respondent at their health facility. Under the guidance of the researcher and study assistants, the literate CBMs completed the questionnaires. Research assistants read the questionnaire responses in the local language to illiterate mothers.

Statistical analysis

We checked the completed questionnaire copies to ensure all answers had been completed appropriately. Copies of the questionnaire with missing data were not included in the analysis. SPSS Version 25 (IBM Corp., Armonk, NY, USA) was used to analyze the data. For data analysis, inferential as well as descriptive statistics were used. While inferential statistics such as simple linear regression were used to test the null hypotheses, descriptive statistics such as mean, standard deviations, and point-biserial correlation were employed to address the study objectives. The genuine boundaries of numbers were utilized to ascertain the degree of IMNCH service utilization. The aforementioned suggests that mean (x) scores falling between 0.00 and 1.49 denoted a low level of utilization, 1.50 and 2.49 represented a moderate level of utilization, 2.50 and 3.49 represented a high level of utilization, and 3.50 and 4.00 represented a very high level of utilization.

The Nwagu and Agbaje recommendations for the interpretation of correlation coefficients were applied to understand the direction, strength, and size of the correlations among the variables [16]. Therefore, a correlation coefficient between ±0.00 and ±0.29 was considered to indicate a no-to-weak link (NR/WR); a correlation coefficient between ±0.30 and ±0.59 was considered to indicate a moderate association (MR); and values between ±0.60 and ±0.99 were considered to indicate a strong relationship (SR). At the 0.05 level of significance, the null hypotheses were tested using simple linear regression.

Ethical approval

The Ministry of Health and Human Services, Benue, Nigeria, provided ethical authorization (MOH/STA/204/VOL.1/39). The study goal was communicated to the administrative offices in each facility, and consent was secured. After explaining the goals and advantages of the study, participants gave their informed consent. All data was kept confidential.

## Results

The characteristics of the women who participated in this study (n = 896) are displayed in Table 1. Most participants were unemployed (66.0%) and married (79.7%). Most participants (42.7%) were between the ages of 15 and 24. All respondents had at least a primary education, and 55.5% had completed postsecondary education. Given that 58.9% of the respondents lived in urban areas, there was a nearly equal distribution of respondents (Table 1).

**Table 1:** Sociodemographic characteristics of respondents.

Characteristics	Frequency	Percentage (%)
Place of residence		
Rural area	368	41.1
Urban area	528	58.9
Age group (years)		
15–24	383	42.7
25–34	243	27.1
35–44	259	28.9
45 and above	11	1.2
Parity		
1–2 children	369	41.2
3–4 children	392	43.6
5 children and above	135	15.1
Marital status		
Single	6	0.7
Married	714	79.7
Divorced	152	17.0
Widowed	16	1.8
Separated	8	0.9
Educational level		
Primary education	50	5.6
Secondary education	349	39.0
Tertiary education	497	55.5
Religion		
Christianity	864	96.4
Islam	26	2.9
African Traditional Religion (ATR)	6	0.7
Employment status		
Employed	305	34.0
Unemployed	591	66.0

We noted overall high utilization of IMNCH services among childbearing mothers (x = 3.30, SD = 0.94). Specifically, there was a high utilization of family planning (x = 3.25, SD = 0.94), ANC services (x = 3.34 SD = 0.92), delivery care services (x = 3.39, SD = 0.93), postnatal care services (x = 3.20, SD = 0.96), newborn care services (x = 3.39, SD = 0.93), and child healthcare services (x = 3.24, SD = 0.94) (Table 2).

**Table 2:** Level of utilization of IMNCH services by CBMs (n = 896). x, 0.00-1.49 = low level of utilization (LLU); x, 1.50-2.49 = moderate level of utilization (MLU); x, 2.50-3.49 = high level of utilization (HLU); x, 3.50-4.00 = very high level of utilization (VHLU). x = mean; SD = standard deviation; IMNCH = integrated maternal, newborn, and child health; CBM = childbearing mother

Items	X	SD	Decision
In the last 12 months, to what extent did you utilize any of the following?			
Family planning			
Screening services for diseases before your first pregnancy	3.09	0.99	HLU
Health education services for the prevention of unwanted pregnancy/birth spacing	3.41	0.89	HLU
Cluster	3.25	0.94	HLU
Antenatal care services			
Tetanus vaccination	3.50	0.84	VHLU
Treatment of malaria (i.e., intermittent treatment for prevention of malaria)	3.49	0.82	HLU
Nutritional education for improvement of fetal/child and maternal nutrition	3.38	0.78	HLU
Prevention of communicable diseases	3.18	0.99	HLU
Blood test for anemia, diabetes, hypertension, or HIV	3.24	1.09	HLU
Abdominal examination	3.31	0.92	HLU
Measurement of weight and height for prevention of gestational overweight/obesity	3.29	0.98	HLU
Cluster	3.34	0.92	HLU
Delivery care services			
Abdominal scan (ultrasonography) and vaginal examination	3.34	0.96	HLU
Delivery in the hospital by skilled birth attendants (i.e., nurses, midwives, doctors)	3.53	0.85	VHLU
Examination of placenta for completeness	3.41	0.97	HLU
Use of sterilized equipment such as scissors/scalpel by birth attendant during delivery	3.41	0.92	HLU
Palpation of the abdomen for stimulation and detection of the baby’s position	3.27	0.95	HLU
Cluster	3.39	0.93	HLU
Postnatal care services			
Checking vital signs and general condition after delivery	3.40	0.94	HLU
Immunization services such as tetanus toxoid.	3.14	1.03	HLU
Health education on food preparation and weaning	3.28	0.99	HLU
Counseling services for family planning during postnatal visit	3.14	0.95	HLU
Visited place of delivery for immunization/check-up within 6 weeks of delivery	3.04	0.93	HLU
Advise on personal and environmental hygiene	3.20	0.92	HLU
Cluster	3.20	0.96	HLU
Newborn care services			
Regular provision of immunization (i.e., routine and supplemental immunization)	3.56	0.87	VHLU
Consumption of an adequate diet for the promotion of exclusive breastfeeding	3.37	0.97	HLU
Instruction on the need for adequate rest and sleep	3.23	1.02	HLU
Sleeping under the insecticide-treated net	3.55	0.85	VHLU
Use of skilled newborn care services	3.27	0.92	HLU
Cluster	3.39	0.93	HLU
Child healthcare services			
Regular clinic checkup/screening	3.24	0.92	HLU
Child’s growth monitoring	3.09	1.01	HLU
Health education on childcare practices for mothers.	3.18	1.00	HLU
Demonstration of oral rehydration therapy for the prevention of diarrhea	3.25	0.91	HLU
Health education on the treatment of minor illnesses such as fever and cough	3.26	0.92	HLU
Nutrition education for mothers on exclusive breastfeeding	3.40	0.86	HLU
Cluster	3.24	0.94	HLU
Grand mean	3.30	0.94	HLU

An analysis of the sociodemographic factors showed a positive weak relationship between IMNCH utilization and age (r_bp_ = 0.06, ρ = 0.20), place of residence (r_bp_ = 0.18, ρ = 0.00), parity (r_bp_ = 0.06, ρ = 0.03), marital status (r_bp_ = 0.03, ρ = 0.36) and employment status (r_bp_ ​​​​​​​= 0.02, ρ = 0.03). There is a negative weak relationship between IMNCH utilization and level of education (r_bp_​​​​​​​ = -0.00, ρ​​​​​​​ = 0.03) and religion (r_bp_​​​​​​​ = -0.04, ρ​​​​​​​ = 0.25). This suggests that the sociodemographic factors studied had little or no influence on the utilization of IMNCH services among CBMs (Table 3).

**Table 3:** Point biserial correlation between the utilization of IMNCH and sociodemographic characteristics of CBMs. *r_bp_*, ±0.00 to ±0.29 = no relationship to weak relationship; ±0.30 to ±0.59 = moderate relationship; ±0.60 to ±0.99 = strong relationship. ρ = p-value < 0.05; IMNCH = integrated maternal, newborn, and child health; CBM = childbearing mother

	Sociodemographic variables													
IMNCH services utilization	Age		Place of residence		Parity		Marital status		Educational level		Religion		Employment status	
	r_bp_	ρ	r_bp_	ρ	r_bp_	ρ	r_bp_	ρ	r_bp_	ρ	r_bp_	ρ	r_bp_	ρ
Family planning	-0.01	0.69	0.09	0.01	0.00	0.93	0.13	0.00	0.10	0.00	-0.05	0.13	-0.03	0.40
Antenatal care	0.09	0.01	0.28	0.00	0.06	0.07	0.06	0.07	-0.05	0.14	-0.09	0.01	-0.01	0.77
Delivery services	0.03	0.39	0.15	0.00	0.06	0.09	0.01	0.76	-0.08	0.01	-0.04	0.21	0.09	0.00
Postnatal care	0.11	0.00	0.15	0.00	0.08	0.02	-0.08	0.03	0.03	0.45	-0.03	0.43	-0.06	0.07
Newborn care	0.05	0.12	0.17	0.00	0.13	0.00	0.02	0.62	-0.05	0.16	0.03	0.37	0.09	0.00
Child healthcare	0.3	0.00	0.21	0.00	0.01	0.69	0.01	0.69	0.01	0.88	-0.03	0.36	0.01	0.34
Overall cluster	0.06	0.20	0.18	0.00	0.06	0.03	0.03	0.36	-0.00	0.03	-0.04	0.25	0.02	0.03

Table 4 shows the results of multiple regression analysis of the relationship between IMNCH services utilization and demographic factors of CBMs. The results show that the entire model explained only between 3% and 9% of the variance in the dependent variables (IMNCH services utilization). The table further shows that the place of residence had the most relationship to IMNCH services being significantly related to utilization of family planning services (β​​​​​​​ = 0.316, ρ < 0.05), ANC services (β = 2.662, ρ < 0.05), delivery care services (β = 0.886, ρ​​​​​ < 0.05), postnatal care services (β​​​​​​​ = 1.144, ρ < 0.05), and child healthcare services (β = 1.741, ρ < 0.05). The least related factor to the utilization of IMNCH services was parity and educational level bearing a significant relationship to only newborn services (β​​​​​​​ = 1.032, ρ < 0.05) and family planning services (β ​​​​​​​= 0.341, ρ​​​​​​​ < 0.05), respectively.

**Table 4:** Summary of multiple regression showing the relationship between the utilization of IMNCH services and demographic factors of CBMs. Dependent variables: utilization of family planning services, antenatal care services, delivery care services, postnatal care services, newborn care services, and child healthcare services. Independent variable: sociodemographic factors; ρ (p-value) < 0.05. β = unstandardized coefficient; 95% CI = 95% confidence interval; IMNCH = integrated maternal, newborn, and child health; CBM = childbearing mother

Demographic factors	IMNCH services																	
	Family planning			Antenatal care			Delivery care			Postnatal care			Newborn care			Child healthcare		
	β	95% CI	ρ	β	95% CI	ρ	β	95% CI	ρ	β	95% CI	ρ	β	95% CI	ρ	β	95% CI	ρ
Age	-0.037	-0.161-0.088	0.556	0.229	-0.143-0.427	0.227	0.055	-0.226-0.336	0.700	0.393	0.100-0.683	0.009*	0.193	-0.070-0.455	0.150	0.528	0.197-0.859	0.002*
Place of residence	0.316	0.106-0.526	0.003*	2.662	2.031-3.294	0.000*	0.886	0.409-1.364	0.000*	1.144	0.649-1.640	0.000*	1.032	0.586-1.479	0.000*	1.741	1.179-2.303	0.000*
Parity	0.040	-0.108-0.189	0.595	0.369	-0.080-0.815	0.107	0.274	-0.064-0.612	0.111	0.410	0.059-0.761	0.022*	0.634	0.318-0.950	0.000*	0.060	-0.337-0.458	0.764
Marital status	0.435	0.239-0.632	0.000*	-0.545	-0.046-1.137	0.071	0.013	-0.435-0.461	0.954	-0.561	-1.025- -0.096	0.018*	0.022	-0.396-0.441	0.917	0.507	-0.020-1.034	0.059
Educational level	0.341	0.135-0.547	0.001*	-0.342	-0.943-0.295	0.304	-0.195	-0.662-0.272	0.413	0.214	-0.271-0.698	0.388	0.281	-0.156-0.718	0.207	0.421	-0.129-0.971	0.133
Religion	-0.305	-0.756-0.146	0.184	-1.789	-3.132- -0.426	0.010*	-0.800	-1.826-0.226	0.126	-0.154	-1.218-0.910	0.776	0.340	-0.619-1.299	0.486	-0.272	-1.479-0.935	0.658
Employment status	0.029	-0.210-0.269	0.811	-0.689	-1.407-0.033	0.061	0.434	-0.110-0.978	0.118	-0.393	-0.952-0.172	0.172	0.648	0.140-1.157	0.013*	0.101	-0.539-0.741	0.757
R	0.21			0.31			0.18			0.22			0.23			0.24		
R^2^	0.04			0.09			0.03			0.05			0.05			0.06		

## Discussion

In this study, the utilization of IMNCH services was high. However, utilization was higher with tetanus vaccination, delivery, and childhood immunization. Despite this, we observed that the overall influence of sociodemographic factors on the utilization of IMNCH services in Benue state is minimal. However, the place of residence, educational level, marital status, and parity had a positive relationship with the overall utilization. This finding is similar to other studies that have identified marked utilization of IMNCH services among urban dwellers and CBMs with high educational levels [17,18]. Household barriers to the utilization of IMNCH services exist among married women who may rely on financial support from their husbands, thereby posing barriers to the utilization of these services [19]. Some studies have also shown that men (who are culturally responsible for upholding their local beliefs) often discourage women from utilizing services such as family planning methods in favor of their cultural beliefs [20,21].

Our results, however, identified that the educational level, place of residence, and marital status had a significant relationship with the utilization of family planning services. Education is a long-established determinant of the demand for reproductive healthcare, including self-awareness of the need for reproductive health services [17]. Studies have shown that the limited uptake of modern contraceptive use in Benue State is due to overriding cultural beliefs despite high levels of knowledge and higher educational levels being associated with a better uptake of family planning methods [20,22,23]. Some of the cultural beliefs (in Alekwu and Ibegwu) described include that a woman is married for the purpose of children, a deity would help them in spacing out their children and that contraception amounts to killing the unborn child [20,21]. This situation is worse in rural areas where it is not permitted for a couple to seek contraception [20].

Sparsely distributed health centers limit further access to family planning services, antenatal services, and delivery services among CBMs in rural areas compared to their urban counterparts [18,24]. Our study showed that the place of residence had a significant association with the utilization of family planning, antenatal, delivery, and postnatal services. It has been described that women would walk from place to place (including dispensaries), be unable to find contraceptive medicines, and would have to wait until their next visit to the urban areas to get any [18]. This irregular availability of family planning medications, especially in rural areas, would be detrimental to the timeliness required for the emergency usage of these drugs were necessary. On the other hand, limited utilization of antenatal services due to the place of residence was also identified when NDHS data from 2013-2018 was systematically analyzed [25]. Another study showed that about 25% of women in Benue State spend more than 30 minutes before arriving at their nearest health facility [26]. Women in urban areas enjoy the privilege of a better transport system and a concentration of health facilities, making it easier for them to access health facilities unlike their rural counterparts [26].

ANC services are essential because they ensure a healthy pregnancy for mother and baby, including the transition from labor and childbirth to motherhood. To provide further opportunities to educate pregnant women about their health and that of their children, WHO now recommends a higher frequency of antenatal contacts so that further reductions in perinatal deaths can be achieved [27]. Although this presents an increased cost to CBMs, antenatal service utilization has helped in the early identification of women at an increased risk of pregnancy and childbirth-related complications and has helped them navigate the anticipated problems through apps among other schemes adopted in the community in Benue State [12,13].

Studies have shown that the utilization of delivery services outweighs that of antenatal services in Benue State and other parts of sub-Saharan Africa mostly because of the increased symptomatology and difficulty associated with labor [11]. However, this is met with remarkable challenges as women who choose delivery in healthcare centers would have to travel long distances to access professional healthcare services as these services are concentrated in urban areas far away from laboring pregnant women in rural areas [23]. Finally, inadequate medical logistics such as health personnel, ambulances, and health facilities also hinder women from accessing reproductive healthcare services [17,23]. The implications of this are grave as they reveal an imbalance in resource allocation. In this study, significant associations between age, parity, marital status, employment status, and the utilization of delivery IMNCH services were not found.

Age had a significant influence on the use of postnatal and child healthcare IMNCH services. Age and marital status have been identified as key determinants of the utilization of postnatal care services in other reports [28,29]. Older CBMs aged 25-44 years had a higher utilization of postnatal services compared to their younger counterparts [28,29]. This is because most CBMs around this age would be multiparous and would have had previous exposure to postnatal and child healthcare services and would be familiar with the benefits and the process, unlike younger CBMs. On the other hand, married women were more likely to utilize postnatal services compared to never-married women, which is explained by the presence of financial support from their partners. This finding should, however, be interpreted with caution because unmarried CBMs would find it difficult to identify as unmarried due to the social stigma and scorn associated with having children outside of marriage [29]. Studies have shown that in Nigeria, the likelihood of receiving postnatal care is affected by where they reside. Dwellers in urban residential settlements and living among those who utilized the delivery services were more likely to utilize the postnatal services of IMNCH [29].

Our results suggest that reducing the distance from health facilities will lead to improved access and utilization of IMNCH services among CBMs in Benue State, Nigeria. This is vitally important to reduce maternal mortality in the state. High utilization of delivery services may be linked to increased demand for an acute care service which suggests that this service should be prioritized in the context of improving access to delivery services. Educational status contributes significantly to the utilization of IMNCH services and cannot be ignored. However, further research based on mixed methods might lead to further insights into the contributing factors to the low utilization of IMNCH services in Benue State, Nigeria. Further research is essential to better understand and likely determine the benefit of better and more consistent provisions for educational provisions.

One major strength of this study is the population-based, large sample of tertiary-level hospitals serving rural and urban dwellers with a near-even proportion. This study is limited by the use of a self-reported questionnaire which would present the possibility of an overestimation or underestimation of utilization of IMNCH services that could be aimed at presenting a socially desirable response on the questionnaire. The use of a mixed-method approach would have given more robust results. Second, the cross-sectional study design does not permit causal inferences to be made. However, the findings are an eye-opener to the sociodemographic factors on IMNCH utilization in the study area, which can be leveraged for future studies. Future research is essential in determining the burden of maternal and newborn morbidity and mortality attributable to sociodemographic factors.

## Conclusions

The main determinants identified to be associated with the utilization of IMNCH services include place of residence, age, and marital status. Place of residence was shown to significantly affect all components of IMNCH services which can be attributed to the sparsely distributed healthcare centers in rural areas. These disparities in utilization of IMNCH services affect all the components of IMNCH services and lead to disparities in health outcomes that contribute to the local and national burden of maternal mortality. The next best step would be a strengthened political commitment toward ensuring equitable distribution of health resources to all localities in Benue State, Nigeria.
